# A randomized trial of mail and email recruitment strategies for a physician survey on clinical trial accrual

**DOI:** 10.1186/s12874-020-01014-x

**Published:** 2020-05-19

**Authors:** Caitlin C. Murphy, Simon J. Craddock Lee, Ann M. Geiger, John V. Cox, Chul Ahn, Rasmi Nair, David E. Gerber, Ethan A. Halm, Katharine McCallister, Celette Sugg Skinner

**Affiliations:** 1 Division of Epidemiology, Department of Population & Data Sciences, University of Texas Southwestern Medical Center, 5323 Harry Hines Blvd., Dallas, TX 75390 USA; 2 Department of Internal Medicine, University of Texas Southwestern Medical Center, Dallas, TX USA; 3 Harold C. Simmons Comprehensive Cancer Center, Dallas, TX USA; 4 Healthcare Delivery Research Branch, Division of Cancer Control and Population Sciences, National Cancer Institute, Rockville, MD USA

## Abstract

**Background:**

Patient participation in cancer clinical trials is suboptimal. A challenge to capturing physicians’ insights about trials has been low response to surveys. We conducted a study using varying combinations of mail and email to recruit a nationally representative sample of medical, surgical, and radiation oncologists to complete a survey on trial accrual.

**Methods:**

We randomly assigned eligible physicians identified from the American Medical Association MasterFile (*n* = 13,251) to mail- or email-based recruitment strategies. *Mail-based recruitmen*t included a survey packet with: (1) cover letter describing the survey and inviting participation; (2) paper copy of the survey and postage-paid return envelope; and (3) a web link for completing the survey online. *Email-based recruitment* included an e-mail describing the survey and inviting participation, along with the web link to the survey, and a reminder postcard 2 weeks later.

**Results:**

Response was higher for mail-based (11.8, 95% CI 11.0–12.6%) vs. email-based (4.5, 95% CI 4.0–5.0%) recruitment. In *email-based recruitment*, only one-quarter of recipients opened the email, and even fewer clicked on the link to complete the survey. Most physicians in *mail-based recruitment* responded after the first invitation (362 of 770 responders, 47.0%). A higher proportion of responders vs. non-responders were young (ages 25–44 years), men, and radiation or surgical (vs. medical) oncologists.

**Conclusions:**

Most physicians assigned to *mail-based recruitment* actually completed the survey online via the link provided in the cover letter, and those in *email-based recruitment* did not respond until they received a reminder postcard by mail. Providing the option to return a paper survey or complete it online may have further increased participation in the *mail-based* group*,* and future studies should examine how combinations of delivery mode and return options affect physicians’ response to surveys.

## Background

Patient participation in cancer clinical trials is suboptimal [1], and fewer than half of National Cancer Institute-sponsored trials meet accrual targets [2]. Prior studies have surveyed oncologists about patient barriers to trial participation but have overlooked practice-level barriers that may impede capacity to successfully enroll and care for patients on trials.

Historically, a challenge to capturing oncologists’ insights about trials has been low response rates to surveys [3]. Physician surveys are an important tool for capturing information about the organization and delivery of care, as well as physician knowledge and attitudes [4], often from a representative sample. Response rates vary by survey mode, incentive, and physician characteristics. Mail-based surveys generally yield higher response rates compared to email-based surveys [5, 6], but mail may be costly and have a slower return. More recently, studies have used mixed-modes, or varying combinations of mail and email, to recruit physicians to complete surveys. Mixed-mode recruitment strategies appear to elicit higher response compared to email alone [7, 8]. Few have evaluated the effect of recruitment strategies on response among oncologists.

We conducted a study using varying combinations of mail and email to recruit a national sample of medical, surgical, and radiation oncologists to complete a survey on practice-level barriers to trial accrual. To understand how recruitment strategies affected response, as well as characteristics of the sample, we addressed two research questions:

1. *Does survey response rate differ by recruitment strategy (mail-* vs. *email-based)?*
2. *Are there differences in characteristics of responders* vs. *non-responders?*

## Methods

### Sampling frame

We identified physicians from the American Medical Association (AMA) Physician Masterfile – the standard sampling frame used in national physician surveys because it provides the most complete coverage of the U.S. physician population. We restricted sampling to hematology/oncology specialties (including medical oncology, radiation oncology, and surgical oncology), office-based physicians, age ≤ 75 years, e-mail address on file, and with patient care listed as primary activity (*n* = 13,251).

Our goal was to obtain 1500 survey responses, and we expected a 30% response rate based on comparable reports in the literature [9–13]. Therefore, we stratified eligible physicians by specialty (*n* = 9177 medical oncologists; *n* = 3720 radiation oncologists; *n* = 354 surgical oncologists) to generate a list of 5000 physicians. Because the number of surgical oncologists was much smaller than other specialties, we included all 354 surgical oncologists in the list of 5000 physicians. To obtain the remaining 4646, we used simple random sampling (via PROC SURVEYSELECT without replacement in SAS) to randomly select 2323 medical oncologists and 2323 radiation oncologists. We then randomly assigned all 5000 physicians to a mail- or email-based recruitment strategy. A randomization log was generated by a biostatistician (CA) and sent directly to research staff not involved in the analysis. Study investigators were blind to randomized assignment.

### Recruitment strategies

From May – July 2017, we sent invitations to 5000 physicians to complete a 15-min survey, with questions about: (1) characteristics of physicians’ practices; (2) referral and recruitment of patients to clinical trials; and (3) barriers to trial accrual.

The *mail-based recruitment strategy* included a survey packet with a: (1) cover letter, signed by the Principal Investigator (CSS), describing the survey and inviting participation; (2) telephone number to call for more information or opt-out; (3) paper copy of the survey and postage-paid return envelope; and (4) web link for completing the survey online [14]. To those who had not responded 2 weeks after the survey packet mailing, we sent a reminder postcard with the web link to the online survey. Finally, 2 weeks after the reminder postcard, we mailed a second survey packet to remaining non-responders.

The *email-based recruitment strategy* included an email describing the survey and inviting participation, along with the web link and a unique code to access and complete the survey online [14]. We worked with Medical Marketing Services (MMS, Schaumburg, IL) to deliver emails using the Principal Investigator as the sender name and with the subject line, “Help NCI identify challenges & incentives for oncologists to recruit for trials.” One week after the email, we sent a second email identical to the first. We received reports from MMS after each email with the number of messages delivered, as well as the number of recipients who opened the email and clicked the web link. Finally, 1 week after the second email, we mailed a reminder postcard to physicians who had not yet responded.

Due to low response from the random sample of 5000 physicians, we modified recruitment strategies to invite the remaining 8251 eligible physicians in the sampling frame. We randomly assigned physicians, stratified by specialty (medical oncology and radiation oncology), to a mail- or email-based recruitment strategy using simple random sampling, as above. We recruited these 8251 additional physicians from August – September 2017.

For *mail-based recruitment*, we shortened the cover letter text and changed the signature to the Medical Director of Oncology at our healthcare system (JVC). Because we noted most responders completed the survey online (vs. via paper), we mailed only a second reminder postcard instead of a second survey packet. For *email-based recruitment*, we shortened the subject line and, as we had done previously, sent a second email 1 week after the first and a reminder postcard to non-responders another week later. No other changes were made.

We allowed responses up to 6 months from the first invitation. All survey completers were able to claim a $50 Amazon gift card.

### Statistical analysis

Our primary outcome was response rate, defined as the proportion of invited physicians who returned a survey. In the entire sampling frame (i.e., random sample of 5000 physicians plus remaining 8251), we compared response rate by recruitment strategy (mail- vs. email-based) using a Chi-square test.

We also used a Chi-square test to compare demographic characteristics by recruitment strategy and response (responders vs. non-responders). Demographic characteristics included age, sex, specialty (medical, radiation, surgical oncology), and geographic region (West, Midwest, Northeast, South).

The Institutional Review Board at the University of Texas Southwestern Medical Center determined that this research study (protocol# 092016–096) is exempt in accordance with 45 CFR 46.101(b). In the case of an anonymous survey, completion of the survey is indication of consent to participate. Both the cover letter and email indicated that: participation was voluntary and participants may refuse to answer any questions or stop participation at any time; the survey was anonymous and no personally identifying information would be obtained; and responses would not be traceable back to any participant.

## Results

We invited 13,251 physicians to complete the survey; 6526 were randomized to *mail-based recruitment* and 6725 to *email-based recruitment*. There were no differences in demographic characteristics by recruitment strategy (Table 1).

**Table 1:** Demographic characteristics and response rate by recruitment strategy

	Mail-based recruitment (*n* = 6526)		Email-based recruitment (*n* = 6725)		
	n	%	n	%	*p*-value
Age (years)					0.82
25–34	121	1.9	137	2.0	
35–44	1908	29.2	1921	28.6	
45–54	1959	30.0	2005	29.8	
55–64	1629	25.0	1713	25.5	
65+	909	13.9	949	14.1	
Sex					0.37
Male	4530	69.4	4620	68.7	
Female	1996	30.6	2105	31.3	
Specialty					0.21
Medical oncology	4541	69.7	4630	68.9	
Radiation oncology	1820	27.9	1900	28.3	
Surgical oncology	159	2.4	195	2.9	
Geographic region					0.32
West	1315	20.2	1332	19.8	
Midwest	1452	22.3	1421	21.1	
Northeast	1504	23.1	1570	23.4	
South	2255	34.6	2402	35.7	
Response rate	770	11.8	302	4.5	< 0.01
Response mode (*n* = 1072)					
Returned paper survey	194	25.2	0	0.0	
Completed survey online	576	74.8	302	100.0	

NOTE: Column percentages displayed in table

In *mail-based recruitment*, 383 (5.9% of 6526 mailed) survey packets were undeliverable (i.e., returned to our study office). In *email-based recruitment*, messages were delivered to more than 98% of physicians; across all emails delivered, 9.3–13.3% of recipients opened the email, and 0.5–4.7% clicked the web link. Most opens and clicks occurred within 2 days of email delivery.

**Fig. 1:**
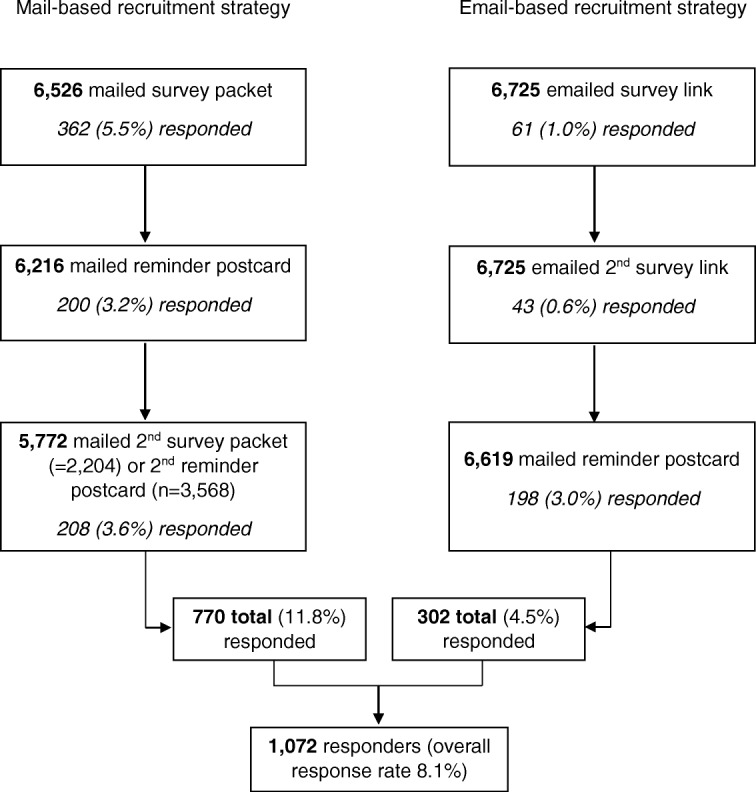
Response to the survey by recruitment strategy

Overall, 1072 (8.1%) physicians responded (Fig. 1). Median time from first invitation to response was 18 days for the *mail-based* and 23 days for the *e-mail based recruitment* strategies. Response rate was higher in *mail-based* (11.8, 95% CI 11.0–12.6%) compared to *email-based* (4.5, 95% CI 4.0–5.0%) *recruitment* (Table 1). Nearly all responders (96.1%, *n* = 1030) completed the survey through to the last question.

As shown in Fig. 1, more physicians in *mail-based recruitment* responded after the first invitation (362 of 770 responders, 47.0%) compared to the second (200 of 770, 26.0%) and third (208 of 770, 27.0%) invitations. About three-quarters (*n* = 576) used the web link included in the cover letter or postcard to complete the survey online instead of completing and returning the paper version (Table 1). For *email-based recruitment*, the largest group of responders (198 of 302 responders, 65.6%) did so after the reminder postcard, mailed 2 weeks after the first email invitation.

Compared to non-responders, a higher proportion of responders were young (ages 25–44 years), male, and radiation or surgical (vs. medical) oncologists (all *p* < 0.05) (Table 2). There was no difference in response by geographic region (*p* = 0.10).

**Table 2:** Differences in demographic characteristics of responders and non-responders, overall and by recruitment strategy (*n* = 13,251)

	Overall (*n* = 13,251)					Mail-based recruitment (*n* = 6526)					Email-based recruitment (*n* = 6725)				
	Responder (*n* = 1072)		Non-responder (*n* = 12,179)		*p*-value	Responder (*n* = 770)		Non-responder (*n* = 5756)		*p*-value	Responder (*n* = 302)		Non-responder (*n* = 6423)		*p*-value
	n	%	n	%		n	%	n	%		n	%	n	%	
Age (years)					< 0.01					< 0.01					< 0.01
25–34	32	12.4	226	87.6		19	15.7	102	84.3		13	9.5	124	90.5	
35–44	406	10.6	3423	89.4		284	14.9	1624	85.1		122	6.4	1799	93.7	
45–54	294	7.4	3670	92.6		217	11.1	1742	88.9		77	3.8	1928	96.2	
55–64	231	6.9	3111	93.1		165	10.1	1464	89.9		66	3.9	1647	96.2	
65+	109	5.9	1749	94.1		85	9.4	824	90.7		24	2.5	925	97.5	
Sex					0.01					0.09					0.03
Male	780	8.5	8370	91.5		555	12.3	3975	87.8		225	4.9	4395	95.1	
Female	292	7.1	3809	92.9		215	10.8	1781	89.2		77	3.7	2028	96.3	
Medical specialty					< 0.01					< 0.01					0.24
Medical oncology	646	7.0	8531	93.0		447	9.8	4100	90.2		199	4.3	4431	95.7	
Radiation oncology	378	10.2	3342	89.8		288	15.8	1532	84.2		90	4.7	1810	95.3	
Surgical oncology	48	13.6	306	86.4		35	22.0	124	78.0		13	6.7	182	93.3	
Geographic region					0.10					0.25					0.40
West	226	8.5	2421	91.5		165	12.6	1150	87.5		61	4.6	1271	95.4	
Midwest	240	8.4	2633	91.7		169	11.6	1283	88.4		71	5.0	1350	95.0	
Northeast	216	7.0	2858	93.0		157	10.4	1347	89.6		59	3.8	1511	96.2	
South	390	8.4	4267	91.6		279	12.4	1976	87.6		111	4.6	2291	95.4	

NOTE: Row percentages displayed in table; *p*-values compare responder to non-responder within each category: overall, mail-based recruitment, and email-based recruitment

## Discussion

Response to the survey on clinical trial accrual was low overall, but our randomized trial yielded several interesting findings. We compared physicians initially recruited to complete the survey by email to those recruited by mail. Response in *email-based recruitment* was 4.5% compared with 11.8% in *mail-based recruitment*. Conventional wisdom suggests email elicits higher response rates because physicians can simply click a web link in an email to access and complete the survey. However, we found the overwhelming majority of physicians assigned to *email-based recruitment* (~ 90%) never opened the email message, and among those who did, only about a quarter clicked the link to open the survey. Even though they were not able to simply click a link to access the survey, most responders in *mail-based recruitment* typed in the web address to complete the online version of the survey (rather than filling out and mailing back the paper copy). Finally, whereas in *mail-based recruitment* most responses were from the first survey packet (i.e., not reminder postcard), in *email-based recruitment*, most responses came after the mailed reminder postcard.

Our finding that response was higher among physicians recruited by mail is consistent with previous studies showing surveys delivered by mail vs. email [7, 8], or a combination of mail and e-mail [5, 6], generally elicit better response than email alone. Most physicians who received mailed invitations responded by typing in the link to complete the survey online. The value of receiving mail invitations was further highlighted by the fact that, in the *email-based recruitment* group, most physicians who completed the survey did so after receiving a mailed postcard reminder.

After low response among the initial 5000 physicians invited to complete the survey, response to email invitations did not improve after attempts to shorten the message and subject line, albeit we did not formally test for differences in response before and after we implemented these changes. The low response in *email-based recruitment* is likely because very few of the recipients (~ 10%) even opened the message, and only about a quarter of those who opened the e-mail even clicked to open the survey. It may be easier to not notice or ignore an e-mail than paper that arrives in one’s physical mailbox. Or the email from an unfamiliar sender may have been delivered to a spam inbox, giving intended recipients no chance to open it. Email recruitment strategies may promise faster response, elicit longer response to open-ended questions [8], and appear as a low-cost alternative to postal mail, but the risk of non-response – especially when the message remains unopened – seems to outweigh the potential benefits of these conveniences [15]. An important avenue of future research is to understand the non-response bias associated with and cost-effectiveness of email recruitment.

A strength of this study was our use of the AMA Physician Masterfile as the sampling frame. The Masterfile offers the most complete coverage of the U.S. physician population because physicians enroll in medical school or during training in the U.S. Nearly all mail and email invitations in our study were delivered, reflecting the accuracy and up-to-dateness of contact information listed in the Masterfile. By inviting all eligible physicians from the Masterfile, we were also able to compare differences in responders vs. non-responders and describe selection bias arising from these differences. Responders were more likely to be younger, male, and surgical (vs. medical or radiation) oncologists. Additionally, the large sampling frame allowed us to achieve a response of more than 1000 surveys, with robust responses to open-ended questions and sufficient sample size to facilitate comparison.

A limitation is that we did not design our study to compare response by survey mode (i.e., online or paper). Specifically, physicians assigned to *mail-based recruitment* were offered a choice to complete the survey online or return by mail, whereas physicians in *email-based recruitment* were provided only a web link to complete the survey online. It was not practical to test two survey modes in *email-based recruitment* unless physicians were instructed to print a survey and return it by mail. We also could not examine the effect on response of making adjustments to invitation length and content.

## Conclusions

In summary, a mail-based recruitment strategy increased physicians’ response to a survey on clinical trial accrual, compared to email-based recruitment. Ironically, most physicians assigned to *mail-based recruitment* actually completed the survey online via the link provided in the cover letter, and those in *email-based recruitment* did not respond until they received a reminder postcard by mail. Providing the option to return a paper survey or complete it online may have further increased participation for those recruited by mail, and future studies should examine how combinations of delivery mode and return options affect physicians’ response to surveys.

## Data Availability

The datasets used and/or analyzed during the current study are available from the corresponding author on reasonable request.

## Abbreviation

AMA: American Medical Association
